# Association of Age with Mortality and Virological and Immunological Response to Antiretroviral Therapy in Rural South African Adults

**DOI:** 10.1371/journal.pone.0021795

**Published:** 2011-07-01

**Authors:** Portia C. Mutevedzi, Richard J. Lessells, Alison J. Rodger, Marie-Louise Newell

**Affiliations:** 1 Africa Centre for Health and Population Studies, University of KwaZulu-Natal, Somkhele, South Africa; 2 Department of Infection and Population Health, University College London, London, United Kingdom; 3 Faculty of Infectious and Tropical Diseases, London School of Hygiene and Tropical Medicine, London, United Kingdom; 4 Institute of Child Health, University College London, London, United Kingdom; Université Paris Descartes, Centre National de la Recherche Scientifique, France

## Abstract

**Objective:**

To assess whether treatment outcomes vary with age for adults receiving antiretroviral therapy (ART) in a large rural HIV treatment cohort.

**Design:**

Retrospective cohort analysis using data from a public HIV Treatment & Care Programme.

**Methods:**

Adults initiating ART 1^st^ August 2004 - 31^st^ October 2009 were stratified by age at initiation: young adults (16–24 years) mid-age adults (25–49 years) and older (≥50 years) adults. Kaplan-Meier survival analysis was used to estimate mortality rates and age and person-time stratified Cox regression to determine factors associated with mortality. Changes in CD4 cell counts were quantified using a piecewise linear model based on follow-up CD4 cell counts measured at six-monthly time points.

**Results:**

8846 adults were included, 808 (9.1%) young adults; 7119 (80.5%) mid-age adults and 919 (10.4%) older adults, with 997 deaths over 14,778 person-years of follow-up. Adjusting for baseline characteristics, older adults had 32% excess mortality (p = 0.004) compared to those aged 25–49 years. Overall mortality rates (MR) per 100 person-years were 6.18 (95% CI 4.90–7.78); 6.55 (95% CI 6.11–7.02) and 8.69 (95% CI 7.34–10.28) for young, mid-age and older adults respectively. In the first year on ART, for older compared to both young and mid-aged adults, MR per 100 person-years were significantly higher; 0–3 months (MR: 27.1 vs 17.17 and 21.36) and 3–12 months (MR: 9.5 vs 4.02 and 6.02) respectively. CD4 count reconstitution was lower, despite better virological response in the older adults. There were no significant differences in MR after 1year of ART. Baseline markers of advanced disease were independently associated with very early mortality (0–3 months) whilst immunological and virological responses were associated with mortality after 12months.

**Conclusions:**

Early ART initiation and improving clinical care of older adults are required to reduce high early mortality and enhance immunologic recovery, particularly in the initial phases of ART.

## Introduction

Older adults (≥50 years old) comprise a significant proportion of people enrolling in HIV treatment programmes in sub-Saharan Africa yet outcomes after initiation of antiretroviral therapy (ART) for this group have not been well described. Older adults have generally been neglected in addressing the global HIV epidemic [1]. Indeed, reporting mechanisms and estimates of epidemiological trends usually only encompass adults aged 15–49 [2]. UNAIDS estimated that globally there were 2.8 million adults aged 50 years and older living with HIV in 2005 [3]. Data from our surveillance programme in rural KwaZulu-Natal estimates overall HIV prevalence rate at 9.5% and incidence of 1% in adults aged 50 years and older [4]. In a verbal autopsy study in rural Kenya, HIV was the cause of death in 27% of people aged 50 years and older and was the leading cause of death up to the age of 70 years [5].

Age is a major determinant of mortality for many diseases in the absence of HIV and ART [6]. In the pre-antiretroviral therapy (ART) era, data from sub-Saharan Africa showed that older age at seroconversion was associated with more rapid progression to death [7], [8], [9], [10]. Since the introduction of ART, there have been conflicting data on outcomes for older individuals. Assessing age as a continuous variable, two studies have suggested an association between increasing age and higher mortality on ART [11], [12]. Two studies analysing age as a categorical variable have reported significantly higher mortality for individuals aged >50 years: the ART-LINC cohort in an analysis of 7160 patients from 10 sites reported a two-fold increased risk in overall mortality for those ≥50 years compared to 16–29 year olds [13]; while in the South African Free State programme there was 58% increased risk of mortality for adults >50 years compared to 20–29 year olds, although the mortality also included people dying before ART initiation [14]. Other studies including a 7 year cohort in Senegal have reported no clear association between age and mortality on ART [15], [16], [17], [18], [19]. Comparison across studies is complicated by the use of different age categories. Moreover these studies have included age as an explanatory variable rather than explicitly assessing mortality within and between younger and older ages. ART outcomes including mortality, immunological and virological response may potentially be influenced by age [20], [21] hence it is important to understand treatment outcomes to inform on appropriate HIV management in older adults. We aim to explicitly assess how mortality rates following ART initiation compare between older and younger adults and the factors associated with mortality in each age category using data from a large rural HIV Treatment and Care cohort and to quantify immunological and virological responses in different age groups.

## Methods

### Ethics statement

Written informed consent was obtained from all participants in the programme to allow use of anonymised routine clinical data in research. Ethical approval for retrospective analysis of these data was obtained from the Biomedical Research Ethics Committee of the University of KwaZulu-Natal (BE066/07) and the Research Office of the KwaZulu-Natal Department of Health.

#### Hlabisa HIV Treatment and Care Programme

The Hlabisa HIV Treatment & Care Programme is a partnership between the local Department of Health (DoH) and the Africa Centre for Health and Population Studies (www.africacentre.ac.za). The details of the programme have been previously described [22], [23].

The programme adheres to the national antiretroviral treatment guidelines which at the time of study recommended initiation of ART for adults with WHO stage IV disease or CD4 cell count ≤200 cells/mm^3^ [24]. Co-trimoxazole was indicated for all individuals with CD4 count ≤200 cells/mm^3^ or WHO stage 3/4. First-line ART consisted of stavudine (d4T), lamivudine (3TC), and either efavirenz (EFV) or nevirapine (NVP). ART was initiated at primary health care (PHC) clinics (or at Hlabisa district hospital) by a physician; monitoring and ART dispensing was subsequently performed by nurses and counsellors. CD4 cell count and HIV viral load were measured every 6 months on ART.

#### Data acquisition

Clinical information at baseline and at monthly clinic visits after initiation of ART is transferred from standardised clinic records to a centralised Microsoft® Access database. A comprehensive tracking service operates whereby patients who are more than one week late for their clinic visit are contacted by telephone and, if necessary, visited at home by a tracker nurse. Information pertaining to death after initiation of ART is therefore obtained either by the clinic staff or tracker team through communication with family members, other clinic staff, or hospital staff. Cause of death is recorded if known but not systematically sought within the routine programme. Laboratory results (CD4 cell count and HIV viral load) are regularly updated from the National Health Laboratory Service (NHLS) laboratory at a district hospital (Hlabisa Hospital). CD4 counts were analysed using the Beckman Coulter EPICS® XL flow cytometer (Beckman Coulter, Inc.). Viral load was measured at a provincial laboratory using the NucliSens EasyQ® HIV-1 assay (bioMérieux), with a lower detection limit of 25copies/ml.

#### Data analysis

Analysis included all adults (≥16 years) who initiated ART between 1^st^ August 2004 and 31^st^ October 2009, excluding patients on ART who transferred into the programme from elsewhere. Analysis was stratified by age at initiation (<50 years and ≥50 years), a classification which ensured consistency with previous reports [21]. The <50 years age group was further stratified into 16–24 years and 25–49 years to assess for heterogeneity in overall outcomes and baseline descriptions. We assessed differences between the three groups in baseline clinical characteristics using the non-parametric equality-of-medians test for continuous variables and proportions test for categorical variables. Estimated glomerular filtration rate (eGFR) was calculated using the 4-variable Modification of Diet in Renal Disease (4-v MDRD) equation, without the ethnicity correction factor, as validated in a South African population [25], [26].

Kaplan-Meier survival analysis was used to assess and compare mortality between and within age strata. Data was censored at earliest of date of death, date of loss to follow-up, date of transfer out of programme, or 22^nd^ April 2010. Loss to follow-up was defined as three consecutive months without a clinic visit. To ascertain the independent influence of age on overall mortality, a Cox regression model adjusted for all significantly different baseline factors (P<0.05) was used to assess mortality hazard difference by age strata. The two bottom age strata (young and mid-age groups) were combined in the analysis for determination of mortality risk factors because there were no statistically significant mortality outcome differences between the two groups. This is also consistent with previous analysis that have assessed those aged below 50 years as one group in comparison to those aged 50 years and above [27], [28], [29], [30]. Stratified Cox regression with time split at 3 and 12 months post-ART initiation was used to determine risk factors for mortality in the periods 0–3 months (very early mortality), 3–12 months (early mortality), and >12 months post-ART initiation. For the two periods in the first year, analysis was further stratified by age to establish differences in mortality predictors between old and young patients. For all Cox models, variables that were associated with mortality at 15% significance level were individually included into the model and model goodness-of fit assessed. Validity of the proportional hazards assumption was tested using the score test based on scaled Schoenfeld residuals [31]. All results are reported at 5% significance level.

Changes in CD4 cell counts in the 24 months following ART initiation were quantified using a piecewise linear model based on follow-up CD4 cell counts measured at six-monthly time points ± three months. For 909 and 504 patients with missing CD4 counts at 6 months and 12 months respectively the value was interpolated from their CD4 cell counts immediately before and after that time point. Of the 2977 patients alive and active 12 months post ART initiation, 2187 patients (73.5%) had a recorded CD4 count.

Virological response at one year was based on viral load measured between 6 and 15 months after ART initiation. The effect of suboptimal virological response (defined as viral load ≥400 copies/ml) on mortality after the first year of ART was quantified in a Cox regression model adjusted for baseline variables and follow-up CD4 cell counts. For both viral loads and CD4 counts, where more than one measurement was available within the specified time period, the one closest to that time point was used.

#### Sensitivity analysis

To account for the effect of missing baseline and follow-up explanatory data, we assessed for any differences in mortality in those with missing observations compared to those with recorded observations. Where those with missing data had significantly different mortality rates, we maintained a category of the missing group within the respective variable in both the univariable and multivariable models exploring factors associated with mortality. This adjusted for any overestimation of the effect of measured/recorded variables on mortality in the absence of those with unmeasured/missing variables. To assess for the extent of loss to follow up bias, we conducted sensitivity analyses where patients lost were considered dead. All analyses were performed with STATA version 11.0 (College Station, Texas, USA).

## Results

### Patient characteristics

Between 1^st^ August 2004 and 31^st^ October 2009, 8846 adults initiated ART in the programme. Of these, 808 (9.1%) were aged 16–24 years, 7119 (80.5%) were aged 25–49 years and 919 (10.4%) were ≥50 years at time of ART initiation (range 16–83 years). Overall median baseline CD4 cell count was 119cells/µl (IQR 58–174). Older adults had the lowest proportion with CD4 cell count <50cells/µl prior to ART initiation and the highest median CD4 count was amongst those aged 16–24 years (Table 1).

**Table 1:** Baseline characteristics for individuals initiated on ART August 2004 - October 2009 (n = 8846), stratified by age at ART initiation.

Variable	16–24 years			25–49 years			50+years		
	*N*	% ormedian (IQR)	(95% CI)	*N*	% ormedian (IQR)	(95% CI)	*N*	% ormedian (IQR)	(95% CI)
**Age**	808	22 (21–24)		7119	35 (30–40)		919	54 (51–58)	
**Male sex**	107	13.24	10.90–15.58	2504	35.17	34.06–36.28	400	43.5	40.32–46.73
**WHO stage 3 or 4**	328	40.59	37.21–43.98	3435	48.25	47.09–49.41	420	45.70	42.48–48.92
Missing	357	44.18	40.76–47.61	2629	36.93	35.81–38.05	348	37.87	34.73–41.00
**CD4 cell count, cells/µl**									
Median (IQR)	777	133 (69–182)	125.7–144	6827	115 (55–173)	113–118	888	127 (71–177)	122–136
150–200	220	28.31	25.14–31.48	1643	24.07	23.05–25.08	237	26.69	23.78–29.60
100–149	162	20.85	17.99–23.71	1449	21.22	20.25–22.19	221	24.89	22.04–27.73
50–99	139	17.89	15.19–20.59	1431	20.96	20.00–21.93	178	20.05	17.41–22.68
<50	138	17.76	15.07–20.45	1540	22.56	21.57–23.55	138	15.54	13.16–17.93
>200	118	15.19	12.66–17.71	764	11.19	10.44–11.94	114	12.84	10.64–15.04
**Viral load, log_10_ copies/ml**	491	4.38	4.26–4.56	4313	4.40	4.36–4.43	542	4.53	4.43–4.63
**Weight, kg (IQR)**	704	56	54.7–57.1	6262	59.3	59–59.8	814	60	59.1–61
**TB treatment**	171	21.16	18.34–23.98	1581	22.21	21.24–23.17	175	19.04	16.50–21.58
**Haemoglobin <8 g/dL**	76	9.41	7.39–11.42	576	8.09	7.46–8.72	44	4.79	3.41–6.17
Missing	110	13.61	11.25–15.98	914	12.84	12.06–13.62	101	10.99	8.97–13.01
*eGFR ≤60 ml/min/1.73 m^2^	30	3.71	2.41–5.02	854	12.00	11.24–12.75	311	33.84	30.78–36.90
**Missing**	93	11.5	9.3–13.7	725	10.2	9.5–10.9	86	9.4	7.5–11.2
**Albumin <32 g/L**	440	54.46	51.02–57.89	3764	52.87	51.71–54.03	474	51.58	48.34–54.81
Missing	98	12.13	9.88–14.38	767	10.7	10.05–11.49	93	10.12	8.17–12.07

CI, confidence interval; IQR, interquartile range.

*eGFR, estimated glomerular filtration rate: calculated using 4-variable MDRD equation (without ethnicity correction).

### Mortality

There were 997 deaths in 14,778 person-years of follow-up (72 in adults aged 16–24 years; 790 in adults 25–49 years and 135 in adults ≥50 years at ART initiation). The overall mortality rate was 6.75 per 100 person-years (95% confidence interval [CI] 6.34–7.18), significantly higher for ≥50 year old adults (8.69 per 100 person-years, 95% CI 7.34–10.28) than younger adults (6.18 per 100 person-years, 95% CI 4.90–7.78 and 6.55 per 100 person-years, 95% CI 6.11–7.02 in those age 16–24 years and 25–49 years old respectively). Overall, controlling for baseline differences (sex, WHO disease stage, baseline CD4 cell count, haemoglobin, weight, eGFR, education and employment) there was 32% excess mortality risk in patients aged ≥50 years (aHR 1.32, 95% CI 1.09–1.60, *P* = 0.004) compared to those aged 25–49. There were no significant differences in either overall mortality or time stratified mortality rates between those initiating aged 16–24 and those aged 25–49 (Table 2).

**Table 2:** Mortality rates following ART initiation stratified by age at initiation and cohort period time (N = 8846).

Cohort period (years)	Person-time (years)	Failures	Mortality rate	95% CI
**16–24 years**				
0–0.25	186.34	32	17.17	12.14–24.28
>0.25–1	447.76	18	4.02	2.53–6.38
>1–2	340.94	13	3.81	2.21–6.57
>2	190.76	9	4.72	2.45–9.07
**25–49 years**				
0–0.25	1675.67	358	21.36	19.26–23.70
>0.25–1	4166.90	251	6.02	5.32–6.82
>1–2	3478.11	111	3.19	2.65–3.84
>2	2737.47	70	2.56	2.02–3.23
**≥50 years**				
0–0.25	217.64	59	27.11	21.00–35.00
0.25–1	535.77	51	9.52	7.23–12.53
>1–2	445.10	15	3.37	2.03–5.59
>2	355.74	10	2.81	1.51–5.22
**TOTAL**	**14778.19**	**997**	**6.75**	**6.34–7.18**

**Figure 1:**
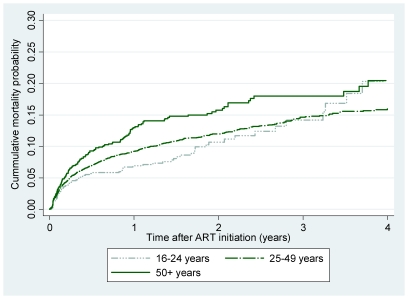
Age and mortality risk post ART initiation. Kaplan-Meier plot of cumulative mortality probability after initiation of ART, stratified by age group at time of ART initiation.

In all age groups, the majority of deaths (769 deaths, 77.1%) occurred in the first year after ART initiation, with mortality particularly high in the first three months after ART initiation (449 deaths, 45.0%). Figure 1A (Kaplan-Meier curve) illustrates mortality differences between the two age groups. Early mortality rates were significantly higher for older adults (≥50 years) but there was no significant mortality difference after 12 months (Table 2).

### Immunological response

**Figure 2:**
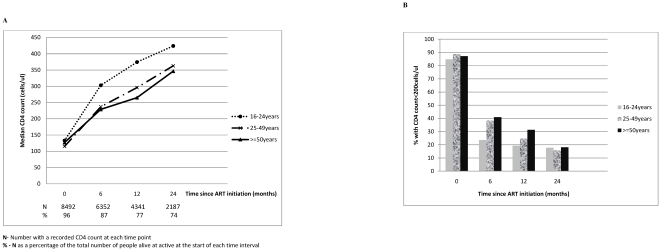
Age and immune response to ART. A. Median CD4 cell count (cells/µl) over time since ART initiation, stratified by age at ART initiation. B. Proportion of patients failing to achieve a CD4 count >200 cells/ul at pre-defined time points post ART initiation, stratified by age at initiation.

Despite baseline CD4 cell count being higher for older adults; their median CD4 cell count post-ART initiation was lower than for both groups of younger adults at each time point (Figure 2A). Overall 16.6% had a poor immunological response (failed to achieve a CD4 count increase of ≥50 CD4 cells) in the first 6 months of therapy with the largest proportion being in those aged 50 years and above (19.6% vs 11.1 and 16.9 in 16–24 year olds and 25–49 years olds respectively). Almost half of all those who initiated with CD4 cell count <50 cells/µl (45.2%) failed to attain a CD4 cell count >200 cells/µl at 12 months. Proportions with CD4 cell counts below 200 cells/µl at specified time points post ART initiation are displayed in Figure 2B.

### Virological suppression

From the 5625 patients recorded as active at 12 months post-ART initiation, 3809 (67.8%) viral loads were available for analysis. Overall 86.3% had a good virological response (<400 copies/ml). A greater proportion of older adults (90.1%, 95% CI 84.7–87.0) had a good response compared to younger adults (81.7%, 95% CI 77.4–86.1 and 86.2%, 95%CI 85.0–87.5 in 16–24 year olds and 25–49 year olds respectively).

### Factors associated with mortality

#### 0–3 months

Using age stratified and time split analysis, from the total 997 deaths, 449 occurred in the first three months after ART initiation (very early mortality) giving the highest period mortality rates of 20.9 and 27.1 per 100 person years in younger and older adults respectively (P = 0.037). However, although mortality risk was significantly higher in the older age group, within each age group, age did not have an independent association with mortality. There was strong evidence of an association between male sex, markers of advanced disease at initiation (CD4 cell count <50 cells/µl, higher log_10_ viral load, lower weight, and albumin <32 g/L) and increased very early mortality in both age groups. In younger adults, but not in older adults, there were additional associations with WHO stage 3/4, low haemoglobin, and renal impairment (Table 3).

**Table 3:** Independent risk factors for very early (0–3 months after ART initiation) and early (3–12 months) mortality stratified by age.

Variable	Very early mortality (0–3 months)		Early mortality (3–12 months)	
	<50 years (n = 7927)	≥50 years (n = 919)	<50 years (n = 7154)	≥50 years (n = 832)
Age (1yr increase)		0.99 (0.95–1.04)		1.03 (0.99–1.08)
25–49 years	1		1	
16–24 years	0.79 (0.54–1.34)		0.73 (0.45–1.19)	
Male sex	1.64 (1.32–2.03)	1.84 (1.06–3.17)	1.40 (1.09–1.80)	1.33 (0.73–2.41)
WHO stage 3 or 4	1.77 (1.11–2.81)	NS	2.06 (1.19–3.57)	NS
CD4 cell count (cells/μl)				
150–200	1	1	1	1
100–149	1.22 (0.79–1.88)	1.03 (0.37–2.86)	1.04 (0.65–1.68)	1.73 (0.70–4.26)
50–99	1.57 (1.05–2.33)	2.34 (0.97–5.67)	1.50 (0.97–2.31)	1.97 (0.79–4.87)
<50	2.38 (1.63–3.46)	2.60 (1.07–6.31)	2.76 (1.85–4.10)	2.00 (0.80–4.98)
>200	1.56 (0.96–2.52)	1.19 (0.35–4.05)	1.50 (0.90–2.51)	2.19 (0.83–5.82)
Missing	2.12 (1.16–3.87)	3.97 (1.10–14.4)	1.80 (0.92–3.51)	0.30 (0.04–2.63)
Viral load (per log_10_ increase)	1.16 (1.03–1.34)	2.28 (1.52–3.43)	NS	NS
Weight (1kg increase)	0.94 (0.93–0.95)	0.96 (0.94–0.99)	0.99 (0.97–1.00)	NS
TB treatment*	1.59 (0.84–1.97)	0.90 (0.48–1.69)	1.05 (0.79–1.40)	1.38 (0.72–2.63)
Haemoglobin <8g/dL	2.06 (1.61–2.64)	NS	NS	4.15 (1.79–9.65)
eGFR ≤60 ml/min/1.73m^2^ †	1.73 (1.35–2.23)	NS	1.41 (1.00–1.98)	NS
Albumin <32g/L	3.58 (2.44–5.24)	2.56 (1.19–5.58)	2.17 (1.56–3.02)	1.52 (0.76–3.02)
missing	4.38 (1.88–10.19)	0.67 (0.42–10.58)	NS	NS

Cox regression models split by time under observation (person years) into very early mortality (0–3 months) and early mortality (3–12 months). Risk factors determined separately for age groups <50 years and ≥50 years.

All values are adjusted hazard ratios with 95% confidence interval.

NS, not significant in univariable model.

*Concurrent TB treatment at time of ART initiation.

† eGFR, estimated glomerular filtration rate: calculated using 4-variable MDRD equation (without ethnicity correction).

#### 3–12 months

Three hundred and twenty deaths; 269 (12.8%) in younger and 51 (20%) in older adults occurred between 3–12 months (early mortality), mortality rates remaining higher in older compared to younger adults (9.5 vs 5.8 per 100 person years respectively; p = 0.001). Low baseline CD4 cell count (<50 cells/µl) remained independently associated with mortality in those aged <50years, as did WHO stage3/4 disease and low albumin. For older adults the only factor independently associated with mortality in this period was haemoglobin <8 g/dL. There remained a trend towards increased mortality risk with CD4 cell count <50 cells/µl and albumin <32 g/dL but the low numbers of deaths in this period for older adults (n = 51) likely limited statistical power (Table 3).

#### After 12 months

Factors associated with mortality after 12 months were explored in a single model incorporating all ages because of the similar mortality rates in both age strata. As such in the adjusted model (Table 4) mortality risk was not significantly different for older adults compared to younger adults (adjusted hazard ratio [aHR] 1.01, 95% CI 0.66–1.55). There was no longer any evidence of an association with baseline CD4 cell count, but a lower absolute CD4 cell count and a reduced increment at 12 months post ART initiation were both associated with higher mortality.

**Table 4:** Independent predictors of mortality after the first 12 months of ART (N = 5625).

Variable	aHR	95% CI
Age 25–49 years	1	
≥50 years	1.01	0.66–1.55
16–24 years	1.35	0.86–2.14
Male sex	1.95	1.46–2.57
Baseline WHO stage 3/4	2.72	1.49–4.97
Missing	2.62	1.43–4.83
Baseline CD4 cell count (cells/µl)		
150–200	1	
100–149	0.80	0.51–1.25
50–99	1.11	0.72–1.71
<50	1.11	0.70–1.75
>200	0.65	0.38–1.13
Missing	0.46	0.20–1.06
Weight (1 kg increase)	0.98	0.96–0.99
Albumin <32 g/L	1.77	1.27–2.47
CD4 increment at 6months (cells/µl)		
<50	1	
50–99	0.98	0.63–1.51
≥100	0.49	0.29–0.81
Missing	1.33	0.39–4.59
Absolute CD4 count at 6months (cells/μl)		
>350	1	
201–350	1.45	0.81–2.57
≤200	0.91	0.44–1.90
CD4 increment at 12months (cells/µl)		
<50	1	
50–99	0.41	0.23–0.73
≥100	0.46	0.24–0.88
Missing	6.15	1.69–22.38
Absolute CD4 count at 12months (cells/μl)		
>350	1	
201–350	0.81	0.43–1.54
≤200	1.49	0.73–3.03
Viral load at 12months (copies/ml)		
<400	1	
≥400	2.67	1.78–4.02
Missing	1.74	1.26–2.41

aHR, adjusted hazard ratio; CI, confidence interval.

Risk factors determined through Cox proportional hazards regression techniques, assessing mortality after 12 months post ART initiation, conditional on being active on the treatment programme at 12 months.

In all models there was no statistically significant association between mortality and either education or employment.

### Sensitivity analysis

Mortality rates did not differ significantly between those with complete baseline observations compared to those with missing observations. However 116 (6.4%) of 1816 patients alive but with a missing viral load at 12 months subsequently died compared to 112 (2.9%) of 3809 with a recorded viral load (*P*<0.001), whilst 103 (7.1%) of those alive but with a missing CD4 cell count at 12 months post ART initiation died compared to 125 (3.0%) of those with a recorded CD4 count (*P*<0.001), resulting in higher mortality risk in some of these missing categories (Table 4).

Overall loss to follow-up was 12.9%; 11.6% and 6.5% in the 16–24 yrs, 25–49 yrs, and ≥50 yrs age groups respectively (p<0.01). Despite these differences, the sensitivity Kaplan Meier and Cox regression analysis results did not differ significantly from those obtained using completely observed data.

## Discussion

We used a large rural HIV treatment programme in South Africa, with a comprehensive tracking system for patients lost to follow-up, to assess mortality rates and differences in three population groups defined by age. In this analysis of 8846 adults with 997 deaths, overall mortality risk was 32% higher for those who initiated ART at age ≥50 years compared to those initiating at age 25–49. Although consistent with previous reports from urban African settings [13], [14] we show that this mortality difference is only evident in the first year of ART, following which mortality rates in older adults are no longer different from that in younger adults despite only modest CD4 count reconstitution in the older age group. Previous studies from Europe and North America [32], [33], [34], [35] have also reported poorer immunological but better virological responses in older compared to younger adults but have not explored how these may relate to mortality rates in older age groups receiving ART. Our study shows that despite older adults having a lower proportion of individuals achieving good immunological response in the first year on ART, their mortality rate as a group, after 12 months on ART, was similar to that observed in the younger adult group. This finding coupled with the fact that older adults had a higher proportion of individuals achieving optimal viral suppression, might imply that in older adults, the degree of CD4 count reconstitution may matter less once HIV has been suppressed. Mortality was not significantly associated to either education or employment probably because in this population there is not much heterogeneity in socio-economic variables and everyone is poor [22].

The majority of people enrolled in HIV care and treatment programmes in sub-Saharan Africa are younger adults, consistent with prevalence patterns [37]. In this programme, just over 10% of adults who initiated ART during the study period were ≥50 years old. The higher proportion of males is contrary to the treatment programme in general but is consistent with local prevalence data that shows more males being infected later in life hence expected to access care much later than females [4], [23]. Whilst evidence from high-resource settings has suggested that older adults present with more advanced disease [33], [38], [39], our data suggest the opposite with a higher median CD4 cell count and lower proportion with CD4 cell count <50 cells/µl in older adults. The most striking clinical difference between the groups at baseline was the higher proportion of renal dysfunction at baseline, with 37% of older adults having an estimated glomerular filtration rate (eGFR) of ≤60 ml/min/1.73 m^2^. Consistent with the observed decline in GFR with age, this alerts us to the high frequency of renal disease in this setting which is not always detected with serum creatinine measurements alone [40].

In all age groups, the highest mortality rates were in the first three months of ART in line with data previously published from this and other programmes [23], [41], [42], [43], [44]. Very early and early mortality was higher in older adults, although older adults presented for ART initiation with higher CD4 counts than younger adults. High early mortality mainly associated with advanced disease coupled with blunted immunologic response in older adults raises an important question of whether older adults should initiate ART at higher CD4 count threshold compared to younger adults and calls for interventions to encourage early presentation for ART. Older adults may also potentially benefit from enhanced clinical care during initial phases of ART.

The high number of deaths immediately after ART initiation suggests that this mortality is still driven largely by HIV disease itself. However, for older adults, the higher mortality may be explained by higher prevalence of non-HIV conditions such as cardiovascular diseases and diabetes. Unfortunately we were unable to ascertain the cause of death since this information within the programme was extremely limited, with only 42 of 997 deaths (4.2%) attributed to a specific cause. However, research in similar settings has shown mortality in the first year of ART to be caused predominantly by infectious diseases related to immunosuppression with tuberculosis consistently shown to be the leading cause of death across all age groups followed by cryptococcal disease and other infectious diseases [18], [19], [44], [45]. Although in previous analyses we showed that younger age was associated with higher TB incidence in the first three months of ART [36], it could be that TB presentation is different in older adults or that symptoms are less frequently attributed to TB in this group leading to missed diagnoses and mortality [46]. The contribution of immune reconstitution inflammatory syndrome (IRIS) to early mortality remains unclear; a recent meta-analysis, using data from diverse settings across high-, middle- and low-income settings, suggested that IRIS might be responsible for 21% of all deaths after ART initiation [47]. Whether the incidence, presentation or mortality attributable to IRIS is higher in older adults requires further study.

Our study demonstrates that at 12 months, approximately one-quarter of our cohort had CD4 cell count ≤200 cells/µl with the largest proportion and the poorest immunological response in those aged ≥50 years and this was associated with increased risk of subsequent death. Larger CD4 count increases were significantly associated with reduced mortality risk irrespective of recent absolute CD4 count. In addition previous absolute CD4 cell thresholds (CD4 cell count at 6 months after ART initiation) were not associated with mortality although CD4 count increments of greater than 100 cells/µl at this stage decreased mortality risk beyond a year on therapy. This may possibly imply that as long as there is an immune response greater than a certain threshold, the influence of the absolute CD4 cells count on mortality becomes minimal and non-significant. Despite younger adults demonstrating superior immunological responses, they had inferior virological suppression, a finding that supports previous observations [32], [33], [34], [35], and was associated with a nearly threefold increased risk of mortality after the first year on therapy. Hence the increased risk in older adults associated with poorer immunologic response may have been counteracted by the reduced risk associated with superior virological response resulting in equal mortality risk in both age groups after one year of ART. Although it is possible that this lack of mortality difference may be due to limited statistical power, there are also possible reasons why this may be; the fact that these older adults are seen every 30 days by health care personnel when they come to collect ART may mean that they have a better chance of early diagnosis of age driven morbidities and better clinical management of new and existing morbidities hence limiting the effect of age on mortality. Babiker et al previously suggested that the effect of age on mortality could be attenuated in the HAART era if there was proportionately a reduction in mortality in older age groups. As older adults are at higher risk of HIV mortality primarily due to a faster decline in CD4 cell counts, HAART associated increases in CD4 could have a larger impact in reducing mortality in an older population [6].

Our study population is similar to that from many rural public health HIV treatment programmes and therefore our results are likely generalisable to similar settings in sub-Saharan Africa. The large cohort size and high mortality rates have enabled this analysis [23]. A major strength of our programme is the comprehensive tracking system for patients lost to follow-up which ensures that deaths are ascertained contemporaneously, unlike in many other programmes [48], giving us confidence that our mortality rates are representative of the true population mortality rates.

Our study has certain limitations as a retrospective analysis of routine programmatic data; we were hampered by missing results particularly for follow-up CD4 cell counts and viral loads which we attempted to address by interpolation of missing CD4 cell counts. The blunted immunological response in older adults compared to younger adults might have been underestimated because CD4 cell count changes are influenced by survival bias, i.e. individuals with the worst immunological response are more likely to have died. Although we controlled for multiple biological variables in determining factors associated with mortality, there might still be residual confounding by adherence levels or other unmeasured variables.

Extremely high mortality rates in the first year of ART, more so for older adults suggests that strategies to reduce this early mortality need to be implemented and evaluated with a degree of urgency and that the needs of older adults should be considered within these strategies. Medical interventions, particularly intensive screening and treatment for TB and cryptococcal infection should be implemented and evaluated to improve understanding of the epidemiology of these infections in older adults [49]. Better understanding of the current patterns of testing and health care usage amongst older adults will inform on appropriate age-specific interventions. Making HIV services more acceptable for this age group might get them into HIV care at an earlier stage. We have previously shown lower rates of retention in pre-ART care for older adults [50]; with the known association between older age and more rapid CD4 decline, it is necessary to explore alternative care strategies, which might include integration with other chronic disease management or community-based follow-up [51]. Further work is ongoing within our programme to determine the causes of death and the burden of co-morbidities in the older population. Future work is required to evaluate whether more intensive follow-up impacts on mortality for individuals at high-risk of death in the first few months of ART.

Discussion around older adults and the HIV epidemic in sub-Saharan Africa often only focus on the indirect impact of the epidemic. Our finding of higher mortality on ART for older adults compared to younger adults adds to the evidence base pointing to a substantial direct effect of HIV on older adults' health. As we move into the next phase of ART scale-up the challenges of HIV in older people will need to be addressed with more purpose.
